# Microsurgical Removal of Microcatheter in the Middle Cerebral Artery During Resection of an Arteriovenous Malformation Resection

**DOI:** 10.7759/cureus.1164

**Published:** 2017-04-13

**Authors:** Pejma Shazadeh Safavi, Sohum Desai, Daniel Branch, Juan R Ortega-Barnett

**Affiliations:** 1 University of Texas Medical Branch at Galveston; 2 Surgery, The University of Texas Medical Branch; 3 Division of Neurosurgery, University of Texas Medical Branch at Galveston

**Keywords:** arteriovenous malformation, microcatheter, adhesion, catheter, removable

## Abstract

Surgical resection is the current standard of therapy for the treatment of arteriovenous malformation (AVM). Endovascular embolization is commonly used as an adjunct prior to surgical resection because it is believed to reduce the risk of intraoperative bleeding. Embolization has been associated with other complications including visual deficits, vessel perforation, and catheter adhesion. Catheter adhesion in which retained segments are contained within the embolization cast are not necessarily cause for concern, but retention of larger portions may confer an increased risk of thrombus formation. Such cases warrant the removal of the retained segments or the patient may suffer serious complications including death related to cerebrovascular events. In this case report, we describe a unique case of catheter adhesion in which the extension of the feeding catheter and the length of the introducer was left in its entirety down to the entry portion at the level of the groin and later retrieved in its entirety by craniotomy.

## Introduction

Endovascular embolization is often employed as a stand-alone procedure or performed preoperatively followed by surgical therapy in the treatment of intracranial arteriovenous malformation (AVM). Embolization is often used to gradually reduce nidus flow to an AVM, rather than abrupt cessation that may be observed by other operative means [1-3]. Although embolization is a frequently employed procedure used to treat AVM, much debate exists in the current literature as to when it should be utilized [1-5]. Complete surgical resection is the current standard of therapy for AVM, but adjunctive embolization has been recognized in reducing the risk of bleed during surgical resection [1-6]. Adjunctive embolization has also been employed with other therapies for AVM such as stereotactic radiosurgery (SRS) and gamma knife radiosurgery, but some debate exists as to how embolization may affect complication rates in these procedures [2, 4].

Complications relating to embolization procedures are associated with the location and size of the AVM. Complications following endovascular embolization reported in the current literature include visual deficits, vessel perforation, and catheter adhesion [3-4]. Catheter adhesion is of particular concern when it cannot be removed prior to surgical intervention as thrombus formation can occur anywhere along the length of the retained catheter [7]. In this case report, we describe a unique case of catheter adhesion in which the extension of the feeding catheter and the length of the introducer was left in its entirety down to the entry portion at the level of the groin and later retrieved in its entirety by open osteotomy.

## Case presentation

**Figure 1:**
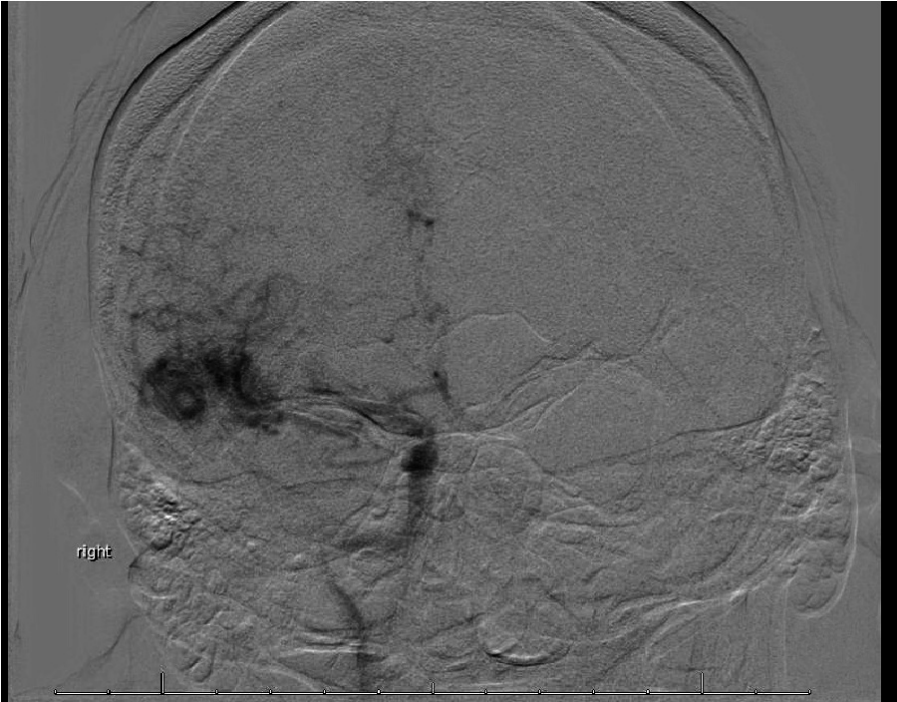
Preoperative Anteroposterior Angiography Anteroposterior Towne's view of early arterial phase angiography demonstrating nidus filling from right middle cerebral artery.

**Figure 2:**
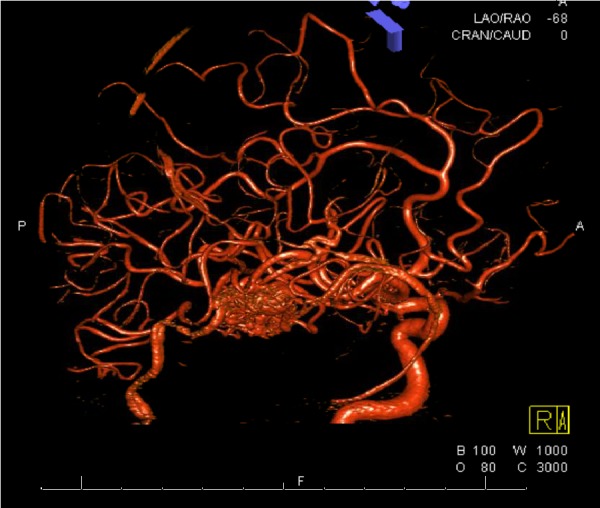
3D Computed Tomography Angiogram Reconstruction 3D - three dimensional

A 38-year-old male with a history of epilepsy presented to us with increasing seizure frequency. His semiology consisted of generalized tonic clonic convulsions. A physical examination revealed that he was neurologically intact with new postictal right lower extremity weakness, which resolved during the hospitalization. T2 weighted MR imaging revealed a 24.2 mm x 16 mm x 13.3 mm roughly ovoid AVM located in the right sylvian fissure. Cerebral angiography demonstrated primary arterial feeders arising from the anterior division of the right middle cerebral artery (MCA) with venous drainage into the right middle cerebral vein and the vein of Labbe (Figure 1). Figure 2 shows a three dimensional (3D) dynamic computed tomography angiogram (CTA) reconstruction of the AVM (Figure 2).

**Figure 3:**
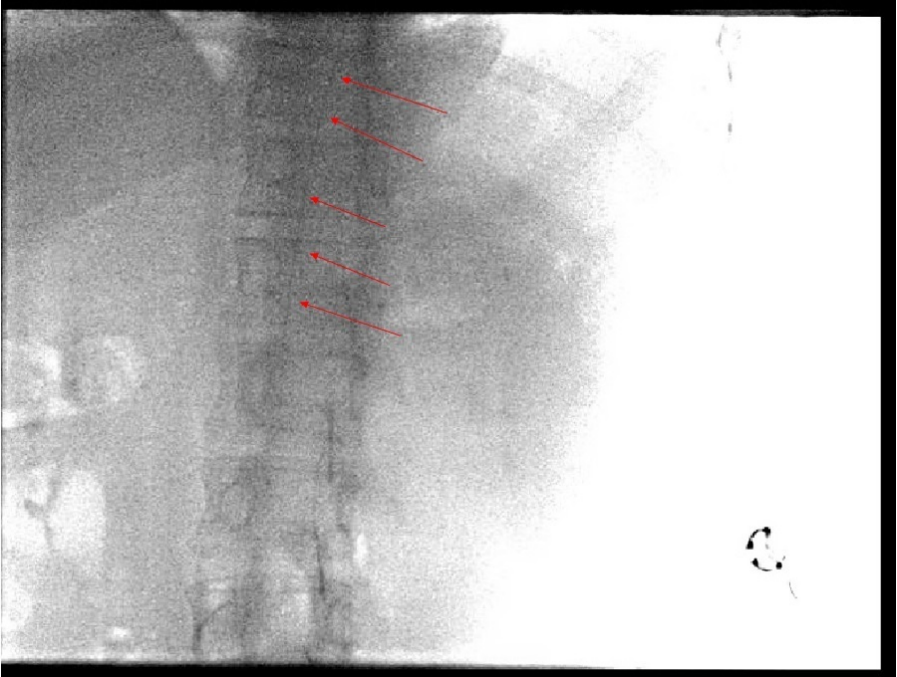
Posterior-Anterior Radiograph Showing Catheter in the Thoracic Aorta (Red Arrows) Coaxial 4.3 French Detachable Apollo Catheter and Marksman Microcatheter in the thoracic aorta.

The patient then underwent endovascular embolization using Onyx 18 (Covidien, Irvine, CA) where near-complete embolization of the AVM was achieved with minimal contrast filling. Towards the end of the procedure and during the attempt to remove the microcatheter, its tip was noted to be adhered to the Onyx cast at the perinidal location. Unsuccessful attempts were made to withdraw the microcatheter by reducing the slack in the system, and traction was then applied over a period of 25 minutes. Given the tortuosity of the vessels and tension noted on the MCA branch, the decision to transect the catheter at the groin site was made to prevent acute intracranial vascular injury. After transection, the distal catheter tip was noted to be positioned along the descending thoracic aorta (Figure 3). Since the patient was scheduled for neurosurgical resection of the AVM the next day, the catheter was removed at that time. The patient was started on the weight-based heparin drip to prevent clot formation along the retained catheter and admitted to the intensive care unit (ICU).

**Figure 4:**
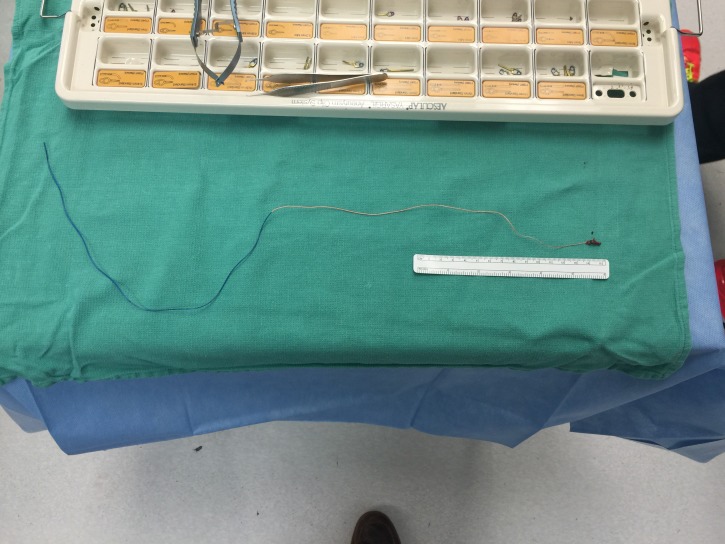
Microcatheter Removed in its Entirety

The following morning, the patient was taken to the operating room where a pterional craniotomy was performed. The sylvian fissure was then split medial to lateral exposing the proximal internal carotid. This was followed distally until visualizing the bifurcation and M1 was readily identified. We could visualize the retained intraluminal catheter at this point. We then turned our attention to the cortical surface where a venous varix was seen. We circumferentially dissected the AVM maintaining venous outflow throughout. Hemosiderin staining around the resection bed suggested prior hemorrhage. We then encountered the two major feeding vessels, which were then bipolared and divided, one of which had the adherent catheter. The entire catheter was then removed (Video 1). Intraoperative angiography was not available. The entirety of the 50 cm catheter was removed during open craniotomy (Figure 4). A follow-up at 12 months revealed that the patient was well with no neurologic deficits.

**Video 1:** Microsurgical Removal of Adhered Microcatheter in the MCA After Embolization of an AVM

## Discussion

Catheter adhesion during embolization is a relatively rare occurrence [8]. The non-adhesive properties of Onyx as an embolization agent, relative to more adhesive agents such as cyanoacrylates, allows for longer injection periods. The use of detachable microcatheters when administering Onyx has been demonstrated to further lengthen injection periods during AVM embolization. Microcatheters may become attached to the extensive retrograde cast of Onyx during the embolization process, leading to vessel injury and, as demonstrated in this case, catheter entrapment [7].

Microsurgical removal of an adherent catheter is risky and not always performed, particularly when the portion of the adherent catheter is limited to the embolization cast and is not likely to be associated with thrombus formation [7-8]. Catheter removal was necessary in this case as the retention of the entire length of the catheter down to the entry point at the groin placed the patient at a significant risk for thrombus development [7]. Heparin therapy kept this patient stable prior to surgical removal of the adherent catheter, and the authors of this case report strongly advise against the use of long term anticoagulant therapy in place of surgical removal in similar cases of catheter adhesion. Surgical removal provides more definitive treatment than long term anticoagulative therapy, and we believe the potential risks, which may include death, associated with retained catheters involving significant portions of the introducer and feeding catheter justify potential risks that may be associated with surgery.

## Conclusions

Endovascular embolization of intracranial arteriovenous malformations can be performed prior to microsurgical resection to minimize the risk of intraoperative hemorrhage, but carries the risk of microcatheter adhesion even when a detachable tip is used.
